# Antibodies against multiple merozoite surface antigens of the human malaria parasite *Plasmodium falciparum *inhibit parasite maturation and red blood cell invasion

**DOI:** 10.1186/1475-2875-9-77

**Published:** 2010-03-18

**Authors:** Ute Woehlbier, Christian Epp, Fiona Hackett, Michael J Blackman, Hermann Bujard

**Affiliations:** 1 Zentrum für Molekulare Biologie (ZMBH), University of Heidelberg, Im Neuenheimer Feld 282, D-69120 Heidelberg, Germany; 2 Division of Parasitology, National Institute for Medical Research, Mill Hill, London NW7 1AA, UK; 3 Current address: Department of Microbiology and Immunology, Virginia Commonwealth University, Richmond, Virginia 23298-0678, USA; 4 Current address: Department of Infectious Diseases, Parasitology, School of Medicine, University of Heidelberg, Im Neuenheimer Feld 324, D-69120 Heidelberg, Germany

## Abstract

**Background:**

*Plasmodium falciparum *merozoites expose at their surface a large protein complex, which is composed of fragments of merozoite surface protein 1 (MSP-1; called MSP-1_83_, MSP-1_30_, MSP-1_38_, and MSP-1_42_) plus associated processing products of MSP-6 and MSP-7. During erythrocyte invasion this complex, as well as an integral membrane protein called apical membrane antigen-1 (AMA-1), is shed from the parasite surface following specific proteolysis. Components of the MSP-1/6/7 complex and AMA-1 are presently under development as malaria vaccines.

**Methods:**

The specificities and effects of antibodies directed against MSP-1, MSP-6, MSP-7 on the growth of blood stage parasites were studied using ELISA and the pLDH-assay. To understand the mode of action of these antibodies, their effects on processing of MSP-1 and AMA-1 on the surface of merozoites were investigated.

**Results:**

Antibodies targeting epitopes located throughout the MSP-1/6/7 complex interfere with shedding of MSP-1, and as a consequence prevent erythrocyte invasion. Antibodies targeting the MSP-1/6/7 complex have no effect on the processing and shedding of AMA-1 and, similarly, antibodies blocking the shedding of AMA-1 do not affect cleavage of MSP-1, suggesting completely independent functions of these proteins during invasion. Furthermore, some epitopes, although eliciting highly inhibitory antibodies, are only poorly recognized by the immune system when presented in the structural context of the intact antigen.

**Conclusions:**

The findings reported provide further support for the development of vaccines based on MSP-1/6/7 and AMA-1, which would possibly include a combination of these antigens.

## Background

**Figure 1:**
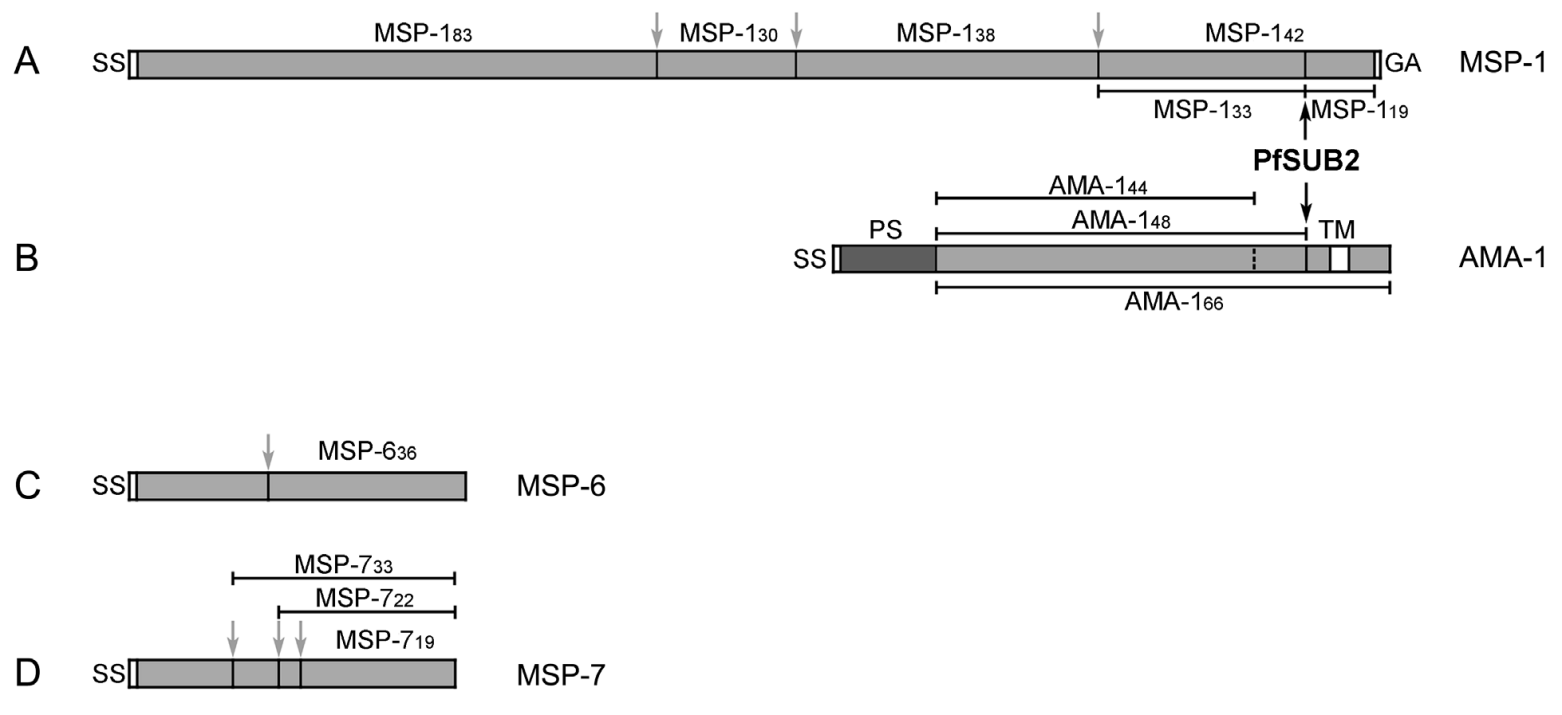
**Primary structure and processing of *P. falciparum *3D7 MSP-1, MSP-6, MSP-7 and AMA-1**. SS, signal sequence; GA, GPI anchor; PS, pro-sequence; TM, transmembrane domain. (A) Outline of the MSP-1 precursor. The grey arrows indicate the sites of primary processing of the precursor protein into its major subunits MSP-1_83_, MSP-1_30_, MSP-1_38_, and MSP-1_42 _as defined by Stafford et al., 1994 [42] and Koussis et al., 2009 [6]. A secondary proteolytic cleavage mediated by PfSUB2 (black arrow) occurs during invasion, cleaving MSP-1_42 _into MSP-1_33 _and MSP-1_19_. (B) AMA-1 is synthesized as an 83 kDa precursor protein containing a C-terminal transmembrane domain (TM). After targeting to the micronemes the N-terminal pro-sequence (PS) is removed, resulting in AMA-1_66_, which appears at the merozoite surface at the time of schizont rupture. During invasion AMA-1_66 _is proteolytically cleaved by PfSUB2 (black arrow) resulting in release of AMA-1_48/44 _[14,15]. MSP-6 (C) and MSP-7 (D) are peripheral merozoite surface proteins, membrane-bound through non-covalent associations with MSP-1. MSP-6 is processed into MSP-6_36_. MSP-7 is initially cleaved into MSP-7_33 _[9]. Around the time of merozoite release from the newly ruptured schizont, MSP-7_33 _is further cleaved into MSP-7_22 _and MSP-7_19 _[9,10].

The severe pathophysiological manifestations of malaria caused by *Plasmodium falciparum *are a direct consequence of the parasite's blood stage replication cycle, during which merozoites repeatedly invade, multiply within, and destroy red blood cells (RBCs). A number of parasite proteins are involved in RBC invasion, of which some, such as MSP-1, MSP-6 and MSP-7, are constitutively exposed at the merozoite surface, while others like apical membrane antigen 1 (AMA-1) are translocated to the merozoite surface only during invasion. All these proteins undergo extensive proteolytic processing at around the point of invasion (Figure 1), and at least two of them - MSP-1 and AMA-1 - are essential in asexual blood-stages [1,2], making them and their maturation potential targets for therapeutic interventions.

MSP-1, which constitutes the major protein component at the merozoite surface [3], is synthesized as a ~190 kDa precursor [4] which is deposited at the parasite plasma membrane *via *a GPI anchor. During the final stages of merozoite maturation, just prior to schizont rupture, MSP-1 is cleaved by a parasite subtilisin-like protease called PfSUB1 into four major subunits, MSP-1_83_, MSP-1_30_, MSP-1_38_, and MSP-1_42_, which remain non-covalently associated [5,6]. The MSP-1 complex interacts with processed forms of MSP-6 and MSP-7, (called MSP-6_36 _and MSP-7_22_) which are thereby peripherally attached to the parasite surface [7-11]. Invasion of RBCs requires a second processing event, which converts MSP-1_42 _into MSP-1_33 _plus a 10 kDa GPI-anchored C-terminal species called MSP-1_19_, which contains tandem epidermal growth factor (EGF)-like domains (Figure 1A). As a result of this processing, the entire MSP-1/6/7 complex is shed from the parasite's surface, except for MSP-1_19 _which is carried into the newly invaded erythrocyte [12].

AMA-1 is initially trafficked as an 83 kDa protein to apical merozoite secretory organelles called micronemes. There, an N-terminal "prosequence" is removed resulting in a 66 kDa processing product called AMA-1_66_. Upon schizont rupture AMA-1_66 _is released from micronemes to become distributed across the merozoite surface, where, at around the point of invasion, it is proteolytically cleaved just upstream of its transmembrane domain. This results in the release of a fragment, comprising the bulk of the AMA-1 ectodomain (called AMA-1_48/44_) from the parasite surface. As a result, only the residual membrane-bound AMA-1 juxtamembrane 'stub' region and associated cytoplasmic domain are transferred into the host RBC [13-15].

Shedding of both MSP-1 and AMA-1 (referred to in both cases as secondary processing) is catalyzed by a second, membrane-bound merozoite subtilisin-like 'sheddase' called PfSUB2, which is released from apical organelles called micronemes onto the merozoite surface at or just prior to invasion [14,16]. For both, MSP-1 and AMA-1, antibodies have been identified which prevent proteolytic processing and RBC invasion *in vitro *[17-20]. In the case of MSP-1, detailed characterization of a panel of monoclonal antibodies targeting conformational epitopes within the C-terminal EGF-like domains revealed that only those that inhibited conversion of MSP-1_42 _into MSP-1_33 _and MSP-1_19 _also prevented invasion [18,21]. Whether antibodies targeting other epitopes within MSP-1/6/7 can block secondary processing and shedding of the complex was hitherto unknown. It is also not known whether antibodies that block shedding of parasite surface proteins by PfSUB2 only affect shedding of their cognate target antigen, or whether they can act in a non-antigen selective manner, thereby suggesting some direct cooperation between these surface proteins during the invasion process.

Recent studies showed that antibodies targeting epitopes throughout MSP-1, MSP-6 and MSP-7 efficiently inhibit parasite growth *in vitro *[11,22]. To gain insight into the mechanisms by which these potentially protective antibodies may act, the effects of these and other invasion-inhibitory antibodies on secondary processing of MSP-1 and AMA-1 have been examined. The current study shows that antibodies targeting numerous epitopes located throughout the multipartite MSP-1/6/7 complex can prevent PfSUB2-mediated secondary processing of MSP-1, suggesting that some antibodies targeting MSP-6 and MSP-7 may act *via *a different mode. Polyclonal antibodies directed against the ectodomain of AMA-1 effectively prevent processing and shedding of AMA-1. Both sets of antibodies appear to function in an entirely specific manner, having no effect on shedding of the other protein(s). MSP-1 epitopes elicit highly inhibitory antibodies, although not as immunogenic in various animal species when presented in the context of the intact antigen. These findings are of relevance for the development of vaccines based on the antigens studied here.

## Methods

### Production of recombinant AMA-1 ectodomain and MSP proteins

The *P. falciparum *FVO AMA-1 corresponding to domains I-III of the ectodomain (Ile^97^-Lys^546^) was expressed in *Pichia pastoris *and purified as described previously [19,23,24]. The MSP-1 (3D7) derived proteins were produced in *Escherichia coli *from synthetic codon optimized DNA sequences as described previously [25]. The MSP-1 complex (p83/30+p38/42) as well as the MSP-1 subunits MSP-1_83_, MSP-1_30_, MSP-1_38 _and MSP-1_42 _were purified as described [26,27]. Production in *E. coli *and purification of N-terminally GST-fused MSP-6 and MSP-7 precursor proteins as well as N-terminally His_6_-tagged MSP-6_36_, MSP-6_36_Δ and MSP-7_22 _was described previously [11,28]. The N-terminally His_6_-fused MSP-1 fragments MSP-1_42ΔEGF _(Ala^1327^-Gln^1612^) and MSP-1_33 _(Ala^1327^-Leu^1606^) were prepared from inclusion bodies which were solubilized in buffer 1 (50 mM Tris, pH 8.0, 4 M guanidine hydrochloride, 3 mM β-mercaptoethanol, 5 mM EDTA) and applied to Ni^2+^-chelate column equilibrated with buffer 1. After washing with buffer 2 (buffer 1 plus 10 mM imidazole), MSP-1_42ΔEGF _or MSP-1_33 _was eluted with buffer 3 (buffer 1 plus 300 mM imidazole). The eluted material was renatured by dialysis against 50 mM Tris, pH 8.0, 1 M arginine, 5 mM DTT, 2 mM EDTA. The MSP-1_19 _fragment (Asn^1607^-Asn^1702^) was produced with an N-terminally fused GST-tag in soluble form and applied to a GSH affinity chromatography in PBS, pH 7.4, 5 mM DTT. After washing with the same buffer p19 was eluted with PBS, pH 7.4, 5 mM DTT, 15 mM GSH. All proteins used herein were buffered in PBS, pH 7.4.

### Antibodies

#### Rabbit antibodies

IgG purified from sera specific for MSP-1_83_, MSP-1_30_, MSP-1_38_, MSP-1_42_, MSP-6 and MSP-7, were described previously [11,22]. The preparation of IgG from pre-immune serum, and of IgG against *Escherichia coli *protein ClpB, used as malaria unrelated control antibody, were described previously [11,22]. For the other antibodies, groups of two or three New Zealand White rabbits were primed on day 0 with 100 μg of purified MSP-1_42ΔEGF_, MSP-1_19_, or MSP-6_36 _in Freund's complete adjuvant (CFA), followed by three boosts on day 28, 42 and 56 with the same amount of protein in Freund's incomplete adjuvant. Serum samples were withdrawn prior to each immunization and two weeks after the last boost total sera were collected. Purified antibody preparations (IgG) from all rabbit sera were obtained by ammonium sulphate precipitation and stored in PBS, pH 7.4, as previously described [22].

#### Mouse sera

Balb/c mice were immunized with purified recombinant AMA-1 ectodomain either in its native state or following reduction and alkylation as described earlier [29] generating 'N' and 'R' sera.

#### Monoclonal antibodies

MSP-1_19 _specific mouse mAb 12.8, 12.10, 7.5, the MSP-1_83 _specific mouse mAb 89.1, and the MSP-1_33 _specific human mAb X509 have all been described previously [5,12], as has been the AMA-1 specific rat mAb 4G2 [19] and the mouse mAb 4F3 (αRAP-1: personal communication, Dr. B. Clough, NIMR, UK).

### Enzyme linked immunosorbent assay (ELISA)

Microtiter plates (96 well) were coated with recombinant protein by overnight incubation with 0.1 ml of 100 nM protein in 50 mM sodium carbonate buffer, pH 9.6 at 4°C. Plates were washed twice with TBST (10 mM Tris-HCl, pH 8.0, 150 mM NaCl, 0.05% Tween 20) before incubation for 1 h at room temperature with blocking buffer (TBST containing 1% milk powder). Antibodies were diluted serially in blocking buffer and incubated with antigen-coated plates for 2 h at room temperature. After three washes with TBST, goat anti-rabbit IgG-alkaline phosphatase conjugate (Sigma, Munich, Germany) diluted 1:2,500 in blocking buffer, was added to the plates and incubated for 1 h at room temperature. The substrate p-nitrophenyl-phosphate (1 mg/ml in 0.96% (vol/vol) diethanolamine, pH 9.5, and 1 mM MgCl_2_) was added, and incubated for 1 h at room temperature in the dark. The reaction was stopped with 0.1 ml of 2 M NaOH, and absorbance was measured at 405 nm. Antibody endpoint titers were calculated as the dilution that produced an absorbance (optical density at 405 nm, OD405) of 1.0. Pre-immune antibody was used as a negative control serum.

### Culturing parasites

*Plasmodium falciparum *3D7 parasites were grown under standard conditions [30] and naturally-released free merozoites were isolated as described [31].

### Processing assays

The effect of antibodies on shedding of AMA-1 and MSP-1 from isolated merozoites was assayed as described [14,31]. Briefly, 3D7 merozoites purified from cultures containing 10 mM EGTA were washed, suspended in 50 mM Tris-HCl, 5 mM CaCl_2 _(pH 7.6) and divided into equal aliquots of approximately 4 × 10^8 ^merozoites, on ice. Aliquots were supplemented with mouse sera, purified rabbit IgGs or monoclonal antibodies, PMSF or control buffers and transferred to 37°C for 2 h. In assays with rabbit antibodies a malaria unrelated control antibody (C) specific for the ClpB protein from *Escherichia coli *was used as a control [31]. Merozoites were pelleted, and shed AMA-1_48+44 _or MSP-1_33 _detected in merozoite supernatants by western blot using mAb 4G2 or mAb X509 respectively.

### SDS-PAGE and western blotting

Parasite lysates were fractionated by SDS-PAGE under non-reducing conditions prior to transfer to Hybond-C extra nitrocellulose membrane (Amersham Biosciences). Membranes were probed with mAbs as previously described [16].

### Parasite growth inhibition assay

Purified rabbit IgGs were examined for their potency in inhibiting *P. falciparum *replication by measuring LDH levels in late trophozoite/early schizont stage cultures as described [22]. Prior to use in growth inhibition assay, rabbit antibody preparations were preadsorbed on type A human RBCs. For this 1 ml of antibody solution was incubated with 50 μl of RBCs (>90% haematocrit) for 1 h. The RBCs were then pelleted by centrifugation, and supernatants were dialyzed against 100 volumes of RPMI medium before use in assays. *Plasmodium falciparum *strain 3D7 was synchronized by magnetic cell separation, cultures were adjusted to 0.4% parasitaemia with human type A erythrocytes at a final haematocrit of 1%, and the final assay volume of cultures was 100 μl containing 25% or 40% (v/v) purified rabbit antibody solution. For monitoring concentration dependencies of antibodies, antibodies were added to the assay in a total volume of 25 μl which contained the respective amount of antibody preparation (3.125 - 25 μl) adjusted to a final volume of 25 μl with pre-immune antibody (PI) whenever required. 25% (v/v) purified pre-immune antibody was used as a control. For monitoring the additive effect of antibodies, antibodies were added to the assay in a total volume of 40 μl, which contained the respective amount of antibody preparation (10 or 20 μl) adjusted to a final volume of 40 μl with pre-immune antibody (PI) whenever required. 40% (v/v) purified pre-immune antibody was used as a control. *Plasmodium falciparum *growth was assayed after 40 h. Inhibition was measured at 650 nm and calculated as follows: % inhibition = 100% - (OD_immune antibody _- OD_RBC_/OD_pre-immune antibody _- OD_RBC_) × 100.

## Results

### Inhibition of MSP-1 secondary processing by MSP-1 specific antibodies

Secondary processing of MSP-1 is readily assayed by monitoring the cleavage of MSP-1_42 _into MSP-1_33 _and MSP-1_19 _in preparations of isolated merozoites [31]. Monoclonal antibodies that prevent this cleavage also inhibit invasion of RBCs by the parasite, as previously demonstrated [32]. Since all these monoclonal antibodies recognize epitopes within MSP-1_19_, it is likely that they act by steric hindrance, preventing access of PfSUB2 to the secondary cleavage site [33]. It was, therefore, of interest to examine whether growth inhibitory antibodies targeting distant regions of MSP-1 also prevent secondary processing, or whether some of them act through a different mechanism.

**Figure 2:**
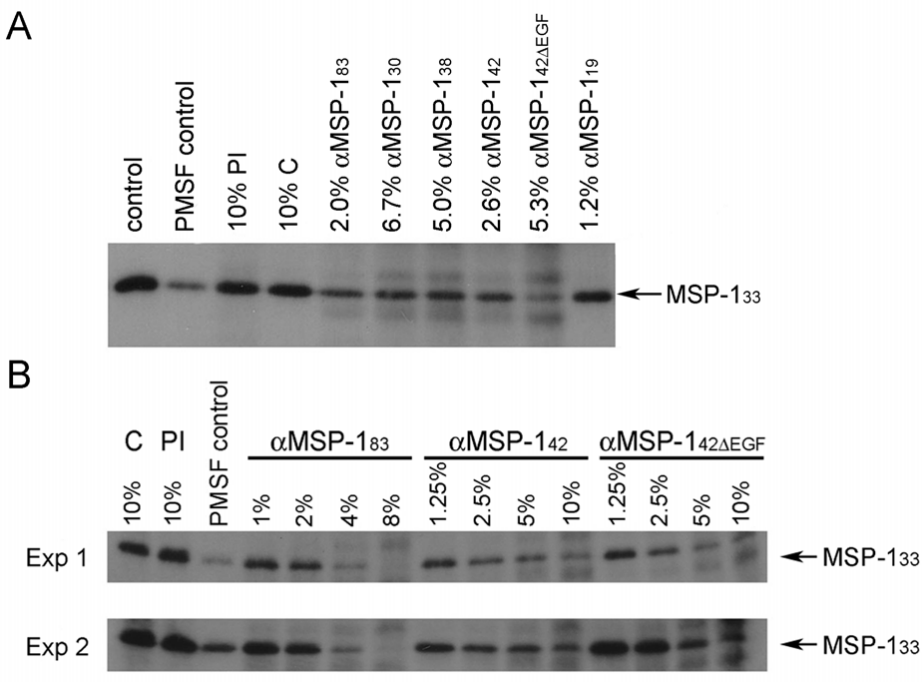
**Inhibition of secondary processing of MSP-1 by antibodies specific for epitopes throughout the MSP-1 molecule**. Secondary processing of MSP-1 in preparations of isolated merozoites was monitored by western blot using mAb X509 to detect the appearance of MSP-1_33 _in merozoite supernatants. (A) Shown is processing of MSP-1_42 _after incubation with medium alone (control), the serine protease inhibitor PMSF (a very effective inhibitor of PfSUB2), pre-immune antibody (PI), malaria unrelated control antibody (C), or with rabbit antibodies specific for various MSP-1 fragments. (B) Shown is the dependency of MSP-1 secondary processing on antibody concentration. Merozoites were incubated in the presence of pre-immune antibody (PI), malaria unrelated control antibody (C), PMSF, or varying amounts of αMSP-1_83_, αMSP-1_42 _or αMSP-1_42ΔEGF _antibodies ranging in concentration from 1% to 10% (v/v) antibody solution. The two sets of panels show western blot results of two different loadings of supernatant samples on SDS-PAGE. The additional bands that appear on all the blots with increasing concentrations of test IgG are due to low-level non-specific recognition of IgG heavy and light chains on the blots by the secondary antibodies used. Loading controls (using mAb 4G2 to detect shedding of the AMA-1 ectodomain) confirmed equal loading in all lanes (data not shown).

To allow for comparisons in this assay, concentrations of antibodies specific for the various MSP-1 fragments were adjusted for maximal invasion inhibition, while still in the range of linear dependency on dilution. All αMSP-1 preparations showed inhibition of secondary processing as judged by visual analysis of the western blot data (Figure 2A), correlating well with their *in vitro *inhibition of parasite multiplication. These findings were confirmed by further experiments in which secondary processing assays were performed using different concentrations of αMSP-1_83_, αMSP-1_42 _and αMSP-1_42ΔEGF _antibodies (Figure 2B). Throughout, levels of processing inhibition correlated with antibody concentration.

### Inhibition of parasite growth in vitro by antibodies specific for MSP-6 and MSP-7

**Figure 3:**
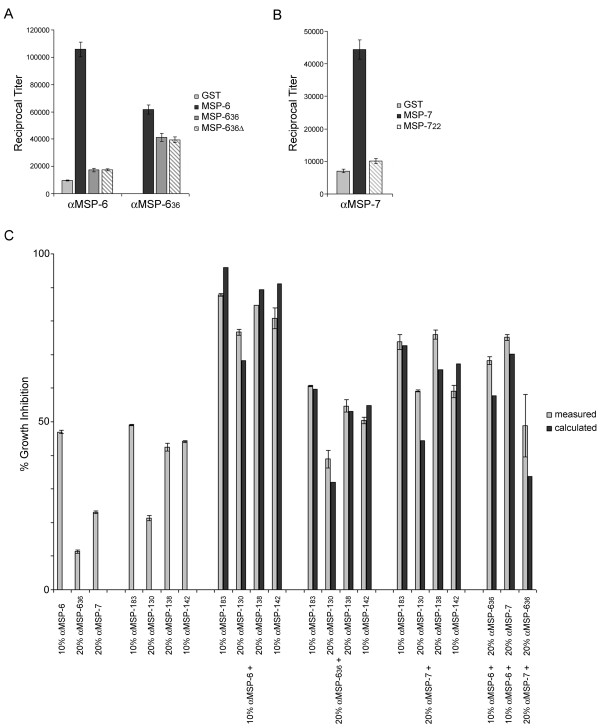
**Inhibition of parasite growth by combinations of antibodies directed towards MSP-1, MSP-6 and MSP-7**. (A) Immunogenicity of recombinant MSP-6 and MSP-6_36 _in rabbits. Endpoint titers were determined against GST (only for αMSP-6, since it was generated using recombinant MSP-6 carrying a GST-tag), MSP-6, MSP-6_36_, and MSP-6_36_Δ. (B) Immunogenicity of recombinant MSP-7 in rabbits. Endpoint titers were determined against GST, MSP-7, and MSP-7_22_. (C) Growth of *P. falciparum *strain 3D7 in the presence of rabbit antibodies specific for MSP-1_83_, MSP-1_30_, MSP-1_38_, and MSP-1_42 _as well as rabbit antibodies specific for MSP-6, MSP-6_36 _and MSP-7. All antibody preparations were diluted to produce between 10-50% inhibition of parasite growth. The indicated combinations were assayed for growth inhibition of *P. falciparum *strain 3D7 via activity of parasite-derived LDH [22]. Antibodies were added to the assay mixtures in a volume of 40 μl (40% of final assay volume) containing 10 μl or 20 μl (10 or 20% of final assay volume) of antibody preparations, as indicated, and whenever necessary adjusted to 40 μl with rabbit pre-immune antibody. Pre-immune antibody (40% v/v) was used as a control. Growth inhibition is presented as percent of control. Dark columns, inhibition calculated from the measured contribution of the individual antibody preparations; light columns, inhibition measured. Error bars show standard deviations derived from triplicate assays. Representative results of one of two experiments are shown.

Antibodies directed against recombinant MSP-6 and MSP-7 inhibit parasite multiplication with comparable efficiency to antibodies raised against MSP-1 subunits, as previously reported [11]. To examine this observation in more detail, rabbit antibodies raised against recombinant MSP-6, MSP-6_36 _and MSP-7, were analysed *via *ELISA using various protein constructs as capture antigen. Immunization with the MSP-6 precursor elicited primarily antibodies directed against the N-terminal part of the molecule not present in the MSP-1/6/7 complex, whereas good titers against the processed product were obtained upon immunization with MSP-6_36 _(Figure 3A). Surprisingly, αMSP-6_36 _antibodies reacted better with the MSP-6 precursor than with the inducing antigen MSP-6_36_. Moreover, when the αMSP-6 and αMSP-6_36 _antibodies were probed against MSP-6_36 _and MSP-6_36_Δ, no difference between the two capture antigens was observed, indicating that the missing C-terminal 51 aa of MSP-6_36_Δ are virtually non-immunogenic in this context.

Immunization with the MSP-7 precursor induced antibodies directed primarily against the N-terminally located processing product, which is not part of the MSP-1/6/7 complex, whereas the C-terminal MSP-7_22 _(Figure 1), when used as capture antigen, revealed only low titers showing its low immunogenicity in the context of the MSP-7 precursor molecule (Figure 3B).

As previously reported [22], combinations of antibodies directed against different parts of MSP-1 have an additive effect on growth inhibition *in vitro*, raising the question of whether combinations of αMSP-6 and αMSP-7 antibodies are additive in their growth inhibitory effect as well. To address this issue, various rabbit antibodies specific for MSP-6, MSP-6_36 _and MSP-7 were combined with antibodies specific for the major MSP-1 subunits as well as with each other and examined for their growth inhibitory potential. As shown in Figure 3C, all combinations demonstrated that the antibodies targeting the various epitopes within the MSP-1/6/7 complex act in an additive manner.

### Inhibition of MSP-1 secondary processing by antibodies specific for MSP-6 and MSP-7

**Figure 4:**
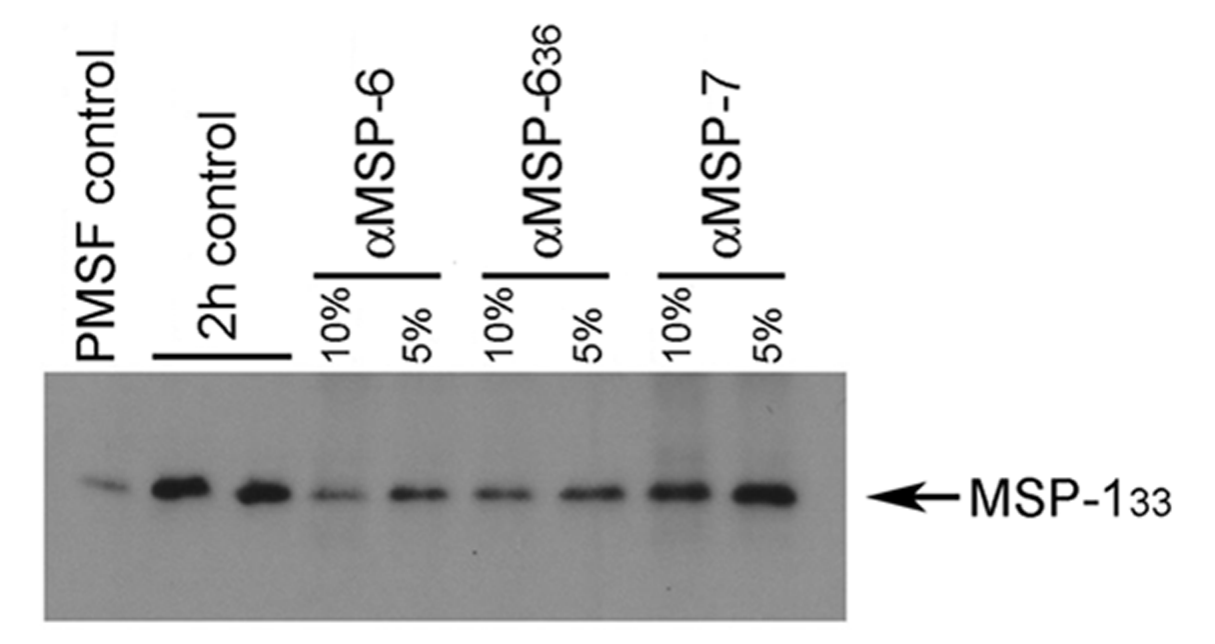
**Inhibition of secondary processing of MSP-1 by antibodies specific for MSP-6 and MSP-7**. Processing of MSP-1_42 _after incubation for 2 h with PMSF, medium alone (2 h control), or medium supplemented with 5 or 10% (v/v) rabbit antibody specific for MSP-6, MSP-6_36 _or MSP-7. Loading controls (using mAb 4G2 to detect shedding of the AMA-1 ectodomain) confirmed equal loading in all lanes (data not shown).

It was of interest to examine whether invasion-inhibitory antibodies targeting MSP-6_36 _and MSP-7_22 _could inhibit secondary processing of MSP-1. As shown in Figure 4, antibodies targeting MSP-6 and its processed form MSP-6_36_, as well as antibodies specific for MSP-7, all reproducibly inhibited MSP-1 secondary processing, confirming that this maturation step is highly sensitive to antibody binding even when the antibodies specifically target the MSP-6 and MSP-7-derived components of the complex.

### Processing-inhibitory antibodies act in a target-specific manner

**Figure 5:**
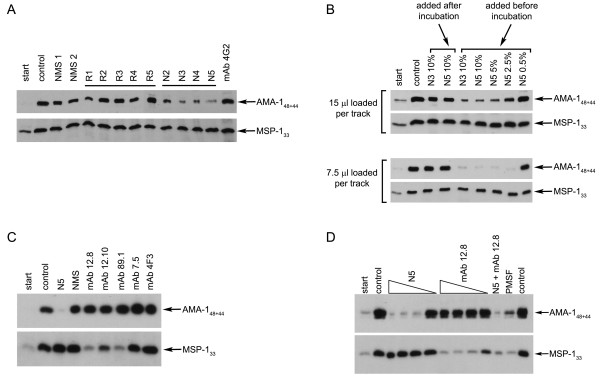
**Specificity of processing inhibition by antibodies targeting MSP-1/MSP-6/MSP-7 or AMA-1**. Processing of AMA-1 and of MSP-1 was detected by western blot using mAb 4G2 and mAb X509 respectively (to identify the processing products AMA-1_44/48 _and MSP-1_33 _respectively). 'Start' indicates samples taken at the beginning of the assay, before incubation of merozoites. (A) Incubation of isolated merozoites in the absence of serum (control), or in the presence of normal mouse sera, (NMS1 and NMS2), or with polyclonal mouse sera raised against reduced and alkylated (R2-R5) or correctly folded (N2-N5) PfAMA-1 ectodomain. Additionally, purified mAb 4G2 was tested for processing inhibition. (B) Dependency of processing on antibody concentration. Merozoites were either incubated in the absence of the sera (tracks 2, 3 and 4 from the left) or in the presence of varying amounts of N3 or N5 serum - presented in terms of percentage (v/v) of serum. The two sets of panels show western blot results of two different loadings of supernatant samples on SDS-PAGE. (C) Processing in presence of AMA-1 antiserum N5, as well as monoclonal antibodies mAb 12.8, mAb 12.10, mAb 7.5 specific for MSP-1_19_, mAb 89.1 specific for MSP-1_83 _and control mAb 4F3. (D) Inhibition of AMA-1 processing by varying amounts of αAMA-1 serum N5, and inhibition of MSP-1_42 _processing by varying amounts of mAb 12.8. Inhibition of processing of both AMA-1 and MSP-1 in the presence of antibodies against both proteins (track 11).

**Figure 6:**
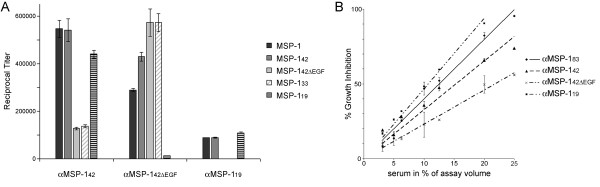
**Inhibition of parasite growth by rabbit αMSP-1 antibodies**. (A) Immunogenicity of MSP-1_42_, MSP-1_42ΔEGF _and MSP-1_19 _in rabbits. Endpoint titers were determined by ELISA against the whole of MSP-1 (in the form of the p83/30+p38/42 heterodimer), MSP-1_42_, MSP-1_42ΔEGF_, MSP-1_33_, or MSP-1_19 _as shown. For the αMSP-1_19 _antibodies the reciprocal titer directed against GST was 22910 +/- 890 (not shown). Error bars show standard deviations. (B) Growth of *P. falciparum *strain 3D7 was monitored in the presence of the indicated antibodies at concentrations ranging from 3.125 to 25% (v/v). Pre-immune antibody (25% v/v) was used as a control. Growth inhibition is presented as percent of control. Error bars show standard deviations.

Shedding (secondary cleavage) of both MSP-1 and AMA-1 from the merozoites surface is mediated by the same protease, PfSUB2 [14,16]. Previous work by others has shown that antibodies against AMA-1 effectively interfere with AMA-1 shedding, and it has been suggested that this may represent one mechanism by which αAMA1 antibodies prevent invasion [17,20]. In view of the above findings, it was explored whether antibodies targeting either the MSP-1/6/7 complex or AMA-1 could affect the processing and shedding of the alternate surface protein. Using mouse sera raised against recombinant AMA-1 ectodomain [19,23], indeed antibodies specific for correctly folded AMA-1 - but not those raised against reduced and alkylated AMA-1 - were found to effectively inhibited processing and shedding of this protein (Figure 5A). However, neither set of sera had any discernible effect on secondary processing of MSP-1 in the same merozoite preparations (Figure 5A, lower panel). Interestingly, no inhibition of AMA-1 processing was observed in the presence of the AMA-1-specific monoclonal antibody 4G2, which contrasts with the findings of Dutta *et al *[17]. Further exploration showed that the effect of αAMA-1 sera was dose-dependent and required their presence during the assay (Figure 5B). Next the most effective αAMA-1 serum (N5) was compared with monoclonal antibodies known to inhibit MSP-1 processing (mAb 12.8, mAb 12.10, mAb 89.1) (Figure 6C). The results confirmed that processing inhibition by these antibodies was exquisitely antigen-specific: N5 serum significantly blocked AMA-1 shedding but had no effect on MSP-1 processing, and, *vice versa*, the αMSP-1 monoclonal antibodies strongly inhibited MSP-1 processing without affecting AMA-1. Again, these effects were dose-dependent, and in the presence of both N5 serum and mAb 12.8, processing of both MSP-1 and AMA-1 was effectively blocked (Figure 5D).

### Inhibition of parasite growth in vitro by MSP-142 specific antibodies

Previous studies [22], examining the growth-inhibitory potential of rabbit antibodies raised against recombinant proteins corresponding to the four primary processing products of MSP-1, revealed not only that antibodies targeting epitopes throughout MSP-1 can effectively interfere with parasite multiplication *in vitro*, but also that the four processing products of MSP-1 differ significantly in their immunogenicity, as reflected by antibody titers in the serum of immunized animals and of individuals living in malaria endemic regions of West Africa [22] and India [28]. The most immunogenic processing product was found to be MSP-1_42_, which also encompasses the secondary processing product MSP-1_19 _(Figure 1A). Immunogenicity did not correlate with the inhibitory potential of the respective antibody preparations [22].

To further dissect the antibody response against MSP-1_42_, the rabbit αMSP-1_42 _antibodies were analysed by ELISA against recombinant full-length MSP-1 [25], MSP-1_42_, MSP-1_33_, and a truncated form of MSP-1_42_, called MSP-1_42ΔEGF_. This latter protein contains the entire cleavage site at the junction between MSP-1_33 _and MSP-1_19 _and an adjacent six amino acids comprising a C-terminal extension into the N-terminus of MSP-1_19_, but it lacks the C-terminal EGF-like domains. The results of these assays (Figure 6A) indicated that by far the most immunogenic region of MSP-1_42 _lies within the MSP-1_19 _portion of the molecule, since about 70% of the αMSP-1_42 _antibodies were found to be directed against this latter region. As expected, the αMSP-1_42ΔEGF _antibodies showed little reactivity with MSP-1_19_, (the low reactivity observed is possibly due to the six amino acid residues shared by the two proteins). However, the αMSP-1_42ΔEGF _antibodies exhibited a high titer against MSP-1_33 _compared to that obtained by immunization with full-length MSP-1_42_. A major fraction of these antibodies presumably targets epitopes, which do not efficiently compete in the context of full-length MSP-1 or MSP-1_42 _(Figure 6A). By contrast MSP-1_19 _alone (fused C-terminally to GST) was significantly less immunogenic, indicating a strong context dependency, which apparently is favourable only when this sequence is embedded in intact MSP-1_42_.

It is well documented that antibodies directed against MSP-1_19 _can efficiently inhibit parasite growth *in vitro*, and that humoral responses against MSP-1_19_-based experimental vaccines can be protective in animal models [34-36]. It was, therefore, of interest to examine whether antibodies raised against MSP-1_42ΔEGF _could inhibit parasite growth *in vitro*. As shown in Figure 6B αMSP-1_42ΔEGF _antibodies indeed inhibited parasite multiplication by 50% when 25% v/v purified antibodies are used in the assay. In comparison αMSP-1_42 _and αMSP-1_19 _reach the same level of inhibition with about 15% and 10% v/v purified antibodies in used in the assay. Relating titers of the antibody preparations shown in Figure 6A with inhibitory potential, the most effective antibodies appear to target MSP-1_19_.

In summary, these analyses support previous findings that multiple epitopes throughout MSP-1 can elicit antibodies, which inhibit parasite growth *in vitro*.

## Discussion

Several lines of evidence indicate that humoral immune responses against *P. falciparum *merozoite surface antigens are major, if not decisive, contributors to the partial immunity acquired by individuals subject to repeated infections. Accordingly, numerous efforts aiming at developing vaccines against *P. falciparum *malaria have focused on such antigens, of which MSP-1 and AMA-1 are particularly promising candidates.

As recently shown [11,22], parasite growth *in vitro *is efficiently inhibited by antibodies targeting different areas of the multipartite MSP-1/6/7 complex, indicating a multitude of epitopes potentially relevant for vaccine development. Such polyclonal antibodies preparations, while primarily preventing invasion of erythrocytes by merozoites, are also capable of inhibiting the intracellular development of the parasite [37]. Moreover, as also revealed in these studies, MSP-1 exhibits an immunogenicity profile in which some epitopes, which induce highly effective inhibitory antibodies, are rather poorly recognized by the immune system as reflected by low titers in serum of mice, rabbits, monkeys and humans.

For vaccine development it is important to not only learn more about relevant epitopes of candidate antigens but also to gain insights into mechanisms by which growth inhibitory antibodies act. Prevention of PfSUB2 mediated secondary processing of MSP-1 and AMA-1, a prerequisite for the shedding of these antigens from the surface of the parasite, has been identified as a plausible mechanism by which inhibitory antibodies targeting these proteins function [17,18,20,38].

Here it was examined whether antibodies specific for the four subunits of MSP-1, MSP-6, or MSP-7 - all of which efficiently inhibit parasite growth *in vitro *- also affect secondary processing and shedding of the complex, or whether additional mechanisms of action may have to be envisioned. The data presented clearly show that antibodies targeting each of the distinct components of the MSP-1/6/7 complex can indeed perturb the function of PfSUB2, and thus are capable of interrupting this important step in the parasite's maturation pathway.

These results are interesting in several regards. Firstly, it would not necessarily be predicted that secondary cleavage, which occurs close to the C-terminus of MSP-1 would be affected by antibodies binding to sites likely to be rather distant from the cleavage site, e.g. within MSP-1_83_, or even within MSP-6 and MSP-7. This may indicate that antibodies bound to the complex may simply sterically hinder access of PfSUB2 to its substrate near the MSP-1 C-terminus. Alternatively, the MSP-1/6/7 complex may constitute a rather dynamic structure sensitive to conformational perturbations, which may occur in any of its domains when antibodies bind. Detailed structure-function studies of the MSP-1/6/7 complex are necessary to provide more insight into either of these hypotheses. Second, as the proteolytic action of PfSUB2 is thought to be a prerequisite for the generation of invasion competent merozoites, it is of interest to note that the MSP-1/6/7 complex obviously offers many targets for antibodies through which the action of PfSUB2 can be inhibited. Indeed, combinations of antibodies targeting different subunits of the complex act in an additive manner. In the context of vaccine development it could, therefore, be of considerable advantage to combine these antigens. Third, it could be shown that inhibitory polyclonal antibodies raised against subunits of the MSP-1/6/7 complex and against the AMA-1 ectodomain respectively, act in a fully specific and independent manner. They interfere neither positively nor negatively with the processing of the "other" protein, suggesting that at least at this stage there is no direct cooperation between these surface proteins. The observation that the invasion-inhibitory αAMA-1 monoclonal antibody 4G2 does not affect processing of AMA-1 is in contrast with the earlier findings of Dutta *et al *[17]. There is no explanation for this discrepancy, but in fact the findings are perfectly in accord with previous data indicating that 4G2 blocks invasion by interfering with interactions between AMA-1 and a partner parasite protein called RON4, implicated in formation of the moving junction between the parasite and host erythrocyte at invasion [39].

When looking at some results described previously [22] and the data reported here in a more quantitative way, two observations deserve discussion in more detail: (i) it appears that some of the most effective MSP-1-specific inhibitory antibodies are elicited by epitopes which are not efficiently presented to the immune system, under natural as well as under laboratory (immunization of laboratory animals with purified antigen) conditions; (ii) antibodies directed against MSP-6 and particularly against MSP-7 are more effective in inhibiting parasite growth than in preventing secondary processing of MSP-1.

To address the first issue, the humoral response against the most immunogenic region of MSP-1 covered by MSP-1_42 _was analysed further. The striking results of these experiments show that epitopes within the highly immunogenic subregion of MSP-1_42_, i.e. within MSP-1_19_, apparently outcompete potentially valuable epitopes located upstream within MSP-1_33_/MSP-1_42Δ*EGF*_. Thus, immunization of rabbits with antigen devoid of the two highly immunogenic C-terminal EGF-like domains elicits high titers of growth inhibitory antibodies, which target regions upstream of MSP-1_19_. These data demonstrate that epitopes within the MSP-1 complex, though not efficiently recognized by the immune system when presented in their natural context, are nevertheless accessible at the surface of the parasite, where the binding of their respective antibodies effectively prevents secondary processing of MSP-1 and thus RBC invasion. Similar constraints likely hold for epitopes located within MSP-1_30 _and MSP-1_38 _[22].

The recognition of the majority of MHC II restricted epitopes depends strongly on structural parameters defined largely by the context in which they are embedded in the antigen [40], whereby local flexibility may play an additional role [41]. Results described here and previously [11,22] have revealed that some of the MSP-1 encoded epitopes which elicit the most effective inhibitory antibodies, are not efficiently recognized by the immune system when presented in the context of the complete antigen, although they are apparently readily accessible to antibodies at the surface of the parasite. The low immunogenicity of such epitopes may actually be one of the reasons why long-lasting, pathogen-clearing humoral responses develop only after repeated exposure to the parasite. For vaccine development it appears therefore important to reconcile particularly such epitopes.

As previously shown [22], polyclonal antibodies specific for the four subunits of MSP-1 inhibit parasite growth not only individually but also when combined, acting in an additive mode. Here, the same holds true when antibodies against MSP-6 and MSP-7 are included in the analysis. In addition antibodies raised against the MSP-6 precursor are more effective inhibitors of secondary MSP-1 processing and of parasite growth, than those induced by MSP-6_36 _(the processed and MSP-1 associated form). These findings suggest that either the MSP-6 precursor more efficiently induces antibodies that bind to MSP-6_36_, or that antibodies directed towards the N-terminal portion of the MSP-6 precursor (not present in the mature MSP-1/6/7 complex) act independently and through a different mechanism. Equally interesting is the observation that antibodies specific for the MSP-7 precursor, which effectively inhibit parasite growth [11], have only a minor effect on the secondary processing of MSP-1, again suggesting further mechanisms of action.

## Conclusions

The present study has yielded a number of insights, which may be summarized as follows: (i) secondary processing of MSP-1, a prerequisite for antigen shedding and subsequent RBC invasion can be inhibited by antibodies targeting numerous epitopes distributed throughout the MSP-1/6/7 complex; (ii) when combined, these antibodies act in an additive manner; (iii) antibodies which prevent PfSUB2 mediated processing of AMA-1 and MSP-1 respectively act independently and do not affect the processing of the alternate protein; (iv) data obtained with MSP-6 and MSP-7 suggest additional mechanisms by which antibodies against the surface proteins studied here may inhibit parasite growth; (v) by dissecting the immunogenicity of MSP-1_42 _further, epitopes were revealed which, in the context of the antigen, do not induce an effective antibody response, even though at the parasite surface they are accessible to their respective antibodies and capable of mediating efficient inhibition of secondary processing and erythrocyte invasion.

The findings presented here provide new insights in the mode of action of antibodies targeting the merozoite surface antigens MSP-1, MSP-6, MSP-7 and AMA-1, and suggest that for optimal vaccine development a more detailed understanding of the structure-function relationship of these antigens is necessary. Equally, these findings provide further support for the feasibility of the development of vaccines or immunization regimens based on these antigens.

## List of abbreviations

α: anti-; AMA-1: apical membrane antigen-1; EGF: epidermal growth factor; GPI: glycosyl phosphatidylinositol; GST: glutathione S-transferase; mAb: monoclonal antibody; MSP: merozoite surface protein; MHC: major histocompatibility complex; pLDH: *Plasmodium *lactate dehydrogenase; PMSF: phenylmethylsulfonyl fluoride; RBC: red blood cell; PfSUB2: *Plasmodium falciparum *subtilisin-like protease.

## Competing interests

The authors declare that they have no competing interests.

## Authors' contributions

UW: Participated in the expression and purification of recombinant antigens of the MSP-1/6/7 complex, and production of rabbit antibodies, designed and performed ELISA and pLDH assays, analysed data and drafted the manuscript. CE: Participated in the expression and purification of the recombinant antigens of the MSP-1/6/7 complex, and production of rabbit antibodies, and assisted in editing the manuscript. FH: Produced mouse antibodies and purified merozoites. MJB: Designed and performed the processing inhibition experiments, analysed data and edited the manuscript. HB: Participated in the study design, planning of the experiments, data analysis, and edited the manuscript. All authors read and approved the final manuscript.
